# Intravenous Tenecteplase for Acute Ischemic Stroke During Active Menstruation

**DOI:** 10.7759/cureus.67186

**Published:** 2024-08-19

**Authors:** Nathan A Baisden, Jordan Preston, Justin Nolte, Jason Adams

**Affiliations:** 1 Neurology, Marshall University Joan C. Edwards School of Medicine, Huntington, USA

**Keywords:** female reproductive, stroke thrombectomy, young adult ischemic stroke, alteplase (tpa), stroke, : ischemic stroke, menses, tenecteplase (tnk)

## Abstract

We report a case of a 51-year-old female who presented to the emergency department with stroke symptoms within the time window for intravenous (IV) thrombolytic therapy. Her initial CT head imaging showed no evidence of acute changes and her CT perfusion demonstrated an area of ischemia in the left parieto-occipital region. While she had no absolute contraindications for IV tenecteplase (TNK), she was actively menstruating at the time, which could represent a relative contraindication due to increased bleeding risk from a site that would not be easily compressible. She elected to receive TNK and did not experience any adverse events after treatment was administered. At her follow-up clinic visit, her neurological deficits were completely resolved.

In the context of increasingly widespread usage of TNK, this case report highlights an uncommon but important consideration when treating acute ischemic strokes with IV thrombolytic in the female population. While no definitive conclusions should be drawn from this case, it would hopefully encourage the continued usage of TNK in menstruating females who present with stroke symptoms within the therapeutic window and with no other contraindications.

## Introduction

While it is well known that recent gastrointestinal hemorrhage and a history of intracranial hemorrhage are contraindications to intravenous (IV) thrombolysis in an acute ischemic stroke setting [1], there is scarce literature on patients who are currently menstruating. Case reports and the original NINDS trial indicate that IV alteplase can be used safely for an acute ischemic stroke in this population [2]. Due to the age at which most females present with strokes, there is a lack of widespread studies on the subject. The most current American Heart Association and American Stroke Association (AHA/ASA) guidelines state that “IV alteplase is probably indicated in women who are menstruating who present with acute ischemic stroke and do not have a history of menorrhagia. However, women should be warned that alteplase treatment could increase the degree of menstrual flow.” [3]

Tenecteplase (TNK) has been recently approved by the AHA/ASA for an acute ischemic stroke [3]; however, little is known about the systemic administration of this agent in females who present within the time window for an acute ischemic stroke and are currently menstruating. The ASSENT-2 trial, which compared outcomes of IV-administered TNK vs. alteplase for acute coronary syndrome (ACS), showed fewer non-cerebral bleeding complications and less need for blood transfusions when using TNK [4]. A subsequent meta-analysis of three clinical trials, again for ACS, demonstrated a statistically significant reduction in major bleeding risk with TNK compared to alteplase [5]. The dosing of TNK for acute ischemic stroke is different than it is for ACS. For ischemic stroke, TNK is dosed at either 0.25 mg/kg (max dose: 25 mg) or 0.4 mg/kg, and for ACS, it is more commonly dosed in weight intervals, but with a maximum dose of 50 mg [3,6]. While the above information is promising for the use of TNK in menstruating females presenting with acute ischemic stroke, it is difficult to extrapolate the ACS data for this specific patient population.

Given the above information, we thought that it would be pertinent to discuss a case of a menstruating female involving the use of IV TNK for an acute ischemic stroke, who met all other criteria for its administration. In this case, TNK was dosed at 0.25 mg/kg (max dose: 25 mg) as previous clinical trials showed no benefit with higher dosing [7]. We hope that this report will encourage continued aggressive usage of this agent in acute ischemic strokes when determined to be of benefit.

## Case presentation

**Figure 1:**
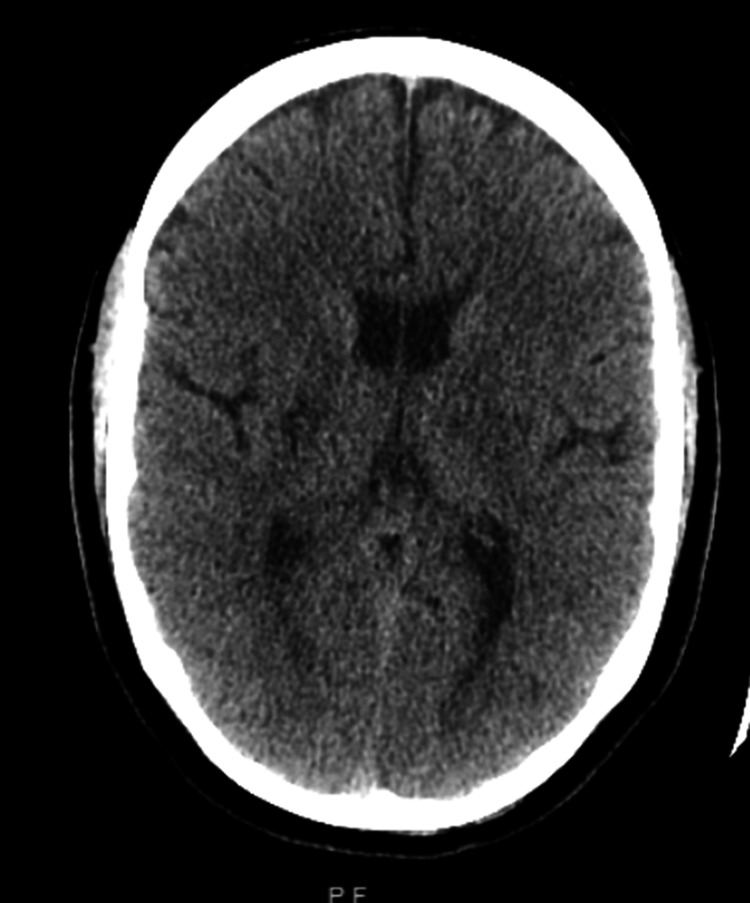
CT head upon presentation The image shows no evidence of intracranial hemorrhage or completed infarct compatible with current symptoms. There was evidence of a previous infarction of the right basal ganglia as reported in prior history CT: computed tomography

**Figure 2:**
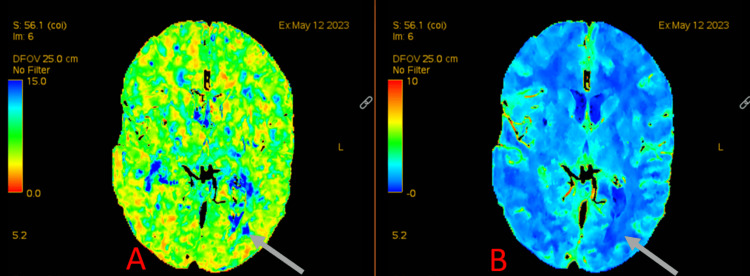
CT angiography perfusion showing mean transit time (A) and cerebral blood volume (B) A. Demonstrates an area of increased mean transit time in the left parieto-occipital region. B. Demonstrates no increased signal on cerebral blood volume imaging in the same area. This indicates an area of ischemic penumbra in this region CT: computed tomography

A 51-year-old female presented to the emergency department as a prehospital stroke alert with right facial droop, right arm and leg weakness, right-sided sensory loss, and dysarthria. Her last known well had been 51 minutes before arrival. The National Institute of Health Stroke Scale (NIHSS) score was 6 and the Modified Rankin Score (MRS) was 4, indicating moderately severe disability. The patient's past medical history was relevant for a history of two previous strokes in the right basal ganglia and right frontal lobe with residual weakness on the left side. Stroke risk factors included hypertension, diabetes, hyperlipidemia, and tobacco usage. Her blood pressure was 190/120 mmHg and her blood glucose was 150 mg/dL. CT head showed no acute changes (Figure 1). CT perfusion showed left parieto-occipital ischemia (Figure 2).

**Figure 3:**
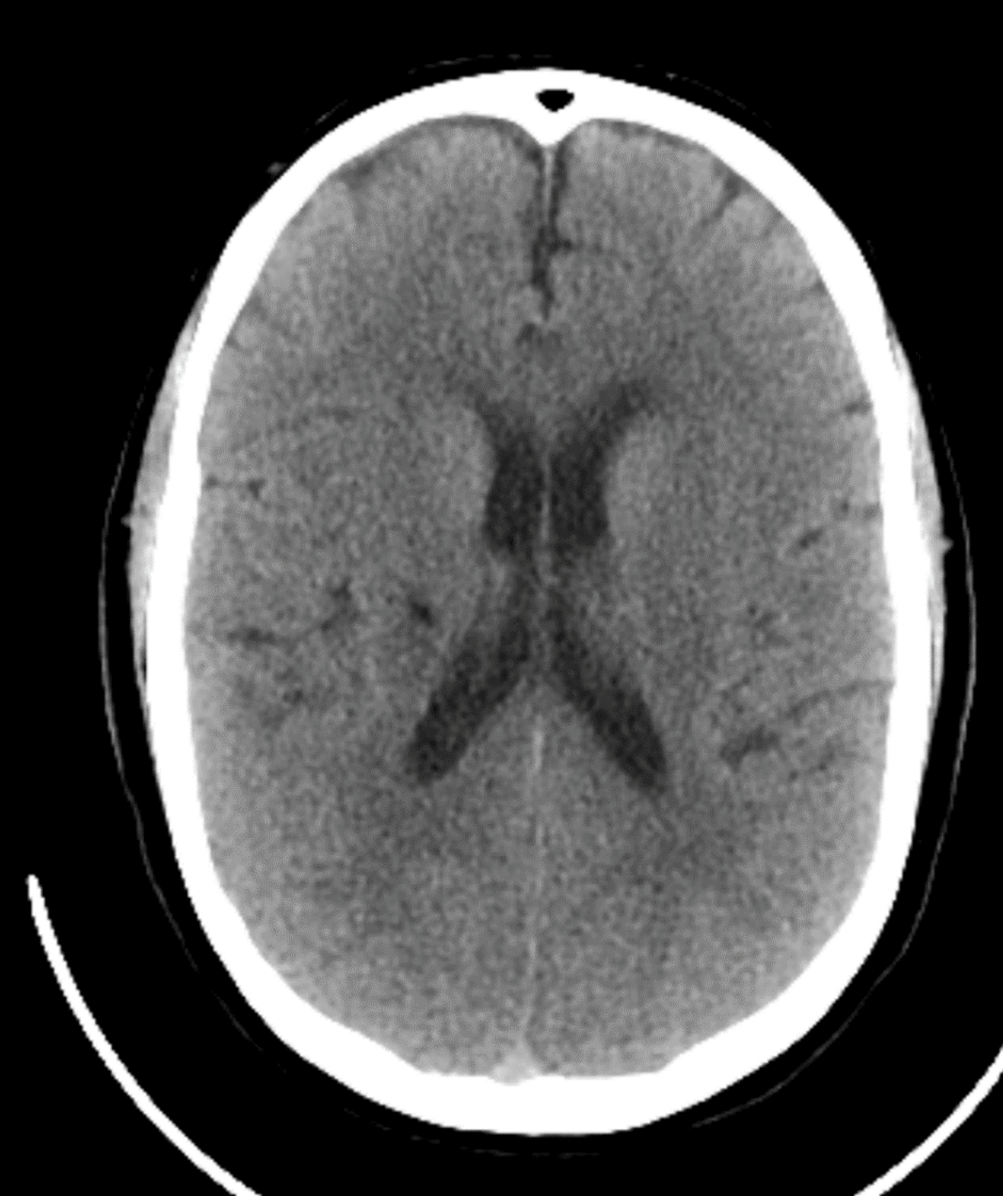
24-hour post-TNK CT head imaging showing no evidence of hemorrhagic transformation CT: computed tomography; TNK: tenecteplase

The patient stated she was menstruating but denied heavy flow or a history of dysfunctional bleeding. Blood pressure remained above the cutoff of 185/110 mmHg for thrombolytic but improved with IV labetalol. The risks and benefits of TNK were discussed, and she elected for treatment despite menstrual bleeding. The patient was not a thrombectomy candidate as there was no evidence of large vessel occlusion on CT angiography; 24-hour post-TNK CT head showed no evidence of hemorrhagic transformation (Figure 3). The patient was subsequently started on aspirin 81 mg daily. No increase in menstrual bleeding was reported by the patient. She showed improvement in her symptoms with NIHSS and MRS of 0 upon discharge to an inpatient rehabilitation facility.

She presented to the neurology clinic for follow-up after discharge from the rehabilitation facility. At her one-month follow-up, she had no right-sided motor or sensory deficits. She did have continued weakness and sensory loss in her left face, arm, and leg consistent with residual deficits from her two prior strokes. She endorsed compliance with aspirin and atorvastatin. Her blood pressure was within normal range, and she reported working with her primary care provider to attain better glycemic control. She continued to smoke cigarettes but endorsed a willingness to work toward cessation. She failed to show up for her three-month post-stroke follow-up and was subsequently lost to follow-up thereafter.

## Discussion

While TNK is increasingly being used for systemic IV thrombolytic therapy of acute ischemic strokes, there is a dearth of data on its use in women who are actively menstruating. Menstruation is not listed as a contraindication for thrombolytic therapy in acute ischemic strokes; however, a recent genitourinary hemorrhage is listed as a relative contraindication [1]. While premenopausal women have an increased risk for stroke [8], it is a rarer phenomenon in menstruating patients. This is likely due to the infrequency of menstruation, occurring approximately every 28 days. It is also known that the risk of stroke increases with age, and hence most ischemic stroke patients are past the point of menstruation [8]. Post-menopausal bleeding is a common phenomenon, but due to time constraints for administering thrombolytic, no distinction was made in this case other than the history that was obtained from the patient. Based on her reporting, the bleeding she was experiencing was a part of her normal menstrual cycle and genitourinary hemorrhaging was ruled out.

TNK is a recombinant DNA-derived version of tissue plasminogen activator which differs from alteplase given its higher affinity for the fibrin component of a thrombus. It also exhibits a greater resistance to inactivation by endogenous plasminogen activator inhibitors, resulting in a longer half-life [9]. These properties are linked to TNK's superior performance over alteplase when treating large vessel strokes in clinical trials [10]. Subsequent analysis showed that TNK was non-inferior to alteplase for all acute ischemic stroke subtypes. Further, TNK was not associated with an increased risk of subsequent symptomatic intracranial hemorrhaging [11]. This, along with the ease of administration in a single bolus, has led to TNK gaining favorability in recent years in the treatment of acute ischemic strokes [12].

Our patient had a history of multiple strokes with residual deficits. It was felt to be in her best interest to treat the current ischemic stroke with thrombolytic therapy as she did not show any evidence of a large vessel occlusion on CT angiography which would have been amenable to mechanical thrombectomy. She was informed of the risk of increased bleeding with systemic TNK but still elected for treatment to prevent further disability. She did not experience any adverse effects and saw the resolution of her right-sided deficits before discharge.

## Conclusions

We discussed a case of a 51-year-old female who was actively menstruating with disabling stroke symptoms and was successfully treated with IV TNK with no reportable side effects and specifically no reported increase in menstrual blood flow. Current genitourinary bleeding is a relative contraindication to IV thrombolysis for an acute ischemic stroke. Shared decision-making between the treating provider and the patient, or their surrogate decision maker, is always encouraged when choosing IV thrombolytic therapy to treat an acute ischemic stroke. While no definitive recommendations can be made based on this case, it would hopefully inspire further analysis on this subject.
